# A Novel Self-Emulsifying Drug Delivery System (SEDDS) Based on VESIsorb^®^ Formulation Technology Improving the Oral Bioavailability of Cannabidiol in Healthy Subjects

**DOI:** 10.3390/molecules24162967

**Published:** 2019-08-16

**Authors:** Katharina Knaub, Tina Sartorius, Tanita Dharsono, Roland Wacker, Manfred Wilhelm, Christiane Schön

**Affiliations:** 1 BioTeSys GmbH, Schelztorstr. 54-56, 73728 Esslingen, Germany; 2 Natural and Economic Sciences, Department of Mathematics, Ulm University of Applied Sciences, Albert-Einstein-Allee 55, 89081 Ulm, Germany

**Keywords:** bioavailability, *Cannabis sativa*, cannabidiol, CBD, hemp extract, human, oral drug delivery system, pharmacokinetic, SEDDS

## Abstract

Cannabidiol (CBD), a phytocannabinoid compound of *Cannabis sativa*, shows limited oral bioavailability due to its lipophilicity and extensive first-pass metabolism. CBD is also known for its high intra- and inter-subject absorption variability in humans. To overcome these limitations a novel self-emulsifying drug delivery system (SEDDS) based on VESIsorb^®^ formulation technology incorporating CBD, as Hemp-Extract, was developed (SEDDS-CBD). The study objective was to evaluate the pharmacokinetic profile of SEDDS-CBD in a randomized, double-blind, cross-over design in 16 healthy volunteers under fasted conditions. As reference formulation, the same Hemp-Extract diluted with medium-chain triglycerides (MCT-CBD) was used. CBD dose was standardized to 25 mg. Pharmacokinetic parameters were analyzed from individual concentration-time curves. Single oral administration of SEDDS-CBD led to a 4.4-fold higher C_max_ and a 2.85-/1.70-fold higher AUC_0–8h_/AUC_0–24h_ compared to the reference formulation. T_max_ was substantially shorter for SEDDS-CBD (1.0 h) compared to MCT-CBD (3.0 h). Subgroup analysis demonstrated a higher bioavailability in women compared to men. This difference was seen for MCT-CBD while SEDDS-CBD mitigated this gender effect. Overall, SEDDS-CBD showed a significant improvement for all determined pharmacokinetic parameters: increased CBD plasma values (C_max_), favorably enhanced bioavailability (AUC) and fast absorption (T_max_). No safety concerns were noted following either administration.

## 1. Introduction

The plant *Cannabis sativa* L. (hemp) comprises a wide variety of phytocannabinoid compounds, including the constituent cannabidiol (CBD) [1]. In recent years, CBD has gained increasing interest due to its various health benefits including antiseizure, analgesic, neuroprotective, anxiolytic, antidepressant, and antipsychotic effects, as well as displaying antioxidative and anti-inflammatory properties [2,3,4,5,6,7]. CBD has a favorable safety and tolerability profile in humans [8,9]. Even high doses of oral CBD do not cause those psychotropic effects that are characteristic for tetrahydrocannabinol (THC) [10].

Cannabinoids are typically consumed by smoking, vaporization, buccal spray or ingested orally in the form of soft gels, oil drops, or cookies [11,12]. The absorption of CBD administered by the mentioned application routes is limited, erratic and results in highly variable pharmacokinetic profiles [13,14,15,16,17,18,19].

Often, oral ingestion of cannabis or cannabis-based products is the preferred route of administration [20,21]. The poor aqueous solubility and extensive first-pass metabolism are thought to be the main reasons for the limited oral bioavailability [2,10,17,22,23]. Furthermore, an effect of food, meaning food/fat-dependent absorption has been shown. Food intake was found to increase area under the curve (AUC) and maximum plasma concentration (C_max_) but also time to reach maximum plasma concentration (T_max_) (delayed absorption) [16]. 

Over the past several years, extensive efforts have been made to improve the oral bioavailability of cannabinoids. Effective formulation strategies include lipid/oil-based formulations [24] and gelatin matrix pellets [2]. More recently, self-emulsifying drug delivery systems (SEDDS) have gained increased interest as an advanced and efficient formulation approach to improve the oral bioavailability of cannabinoids such as THC and CBD [22,23]. Indeed, SEDDS formulation technology resulted in a 4.2-fold higher C_max_ and a 2.2-fold higher AUC of CBD compared to the reference product, the oromucosal spray Sativex^®^. Sativex^®^ is a solution of CBD/THC in ethanol/propylene glycol [23].

SEDDS are mixtures of oils, surfactants and optionally contain hydrophilic solvents. Upon contact with an aqueous phase, such as gastric or intestinal fluids, they spontaneously emulsify under conditions of gentle agitation, similar to those that would be encountered in the gastrointestinal tract. The in situ formed droplets keep the co-administered lipophilic active solubilized in the aqueous environment and enable the transport of the active across the aqueous lumen of the gastrointestinal tract to the surface of the enterocyte, the absorptive epithelium. Single molecules of the active ingredient dissociate from the droplets and are transferred to the enterocyte membrane (flip-flop) and further to the blood or lymphatic vessels. In most cases, SEDDS formulations need to be tailor-made for each active ingredient or mixture of active ingredients. The compositions of SEDDS as well as the resulting droplet size and size distribution formed upon emulsification have been shown to influence the performance of the system regarding bioavailability enhancement [25,26,27,28].

VESIsorb^®^, a self-emulsifying drug delivery formulation technology developed by Vesifact AG (Baar, Switzerland) has shown increased oral bioavailability of lipophilic molecules such as coenzyme Q10 (ubiquinone) [29]. Thus, the objective of the current study was to assess whether a novel SEDDS formulated Hemp-Extract based on VESIsorb^®^ formulation technology (SEDDS-CBD) may improve the oral bioavailability of CBD accordingly. To verify this assumption the pharmacokinetic profile of SEDDS-CBD was evaluated in a single dose (standardized to 25 mg CBD), randomized, double-blind, cross-over study design in 16 healthy volunteers (8 men and 8 women). As reference formulation the same Hemp-Extract diluted with MCT (medium-chain triglycerides) oil (MTC-CBD) was used. To control for confounding factors, especially the possible influence of the described food effect [16] on the pharmacokinetics of CBD, the study was conducted under fasted conditions and strict after dosing diet. Fasting conditions are considered to be the most sensitive conditions to detect a potential difference between formulations.

## 2. Results

### 2.1. Subject Characteristics

The investigated study population was a healthy, non-smoking study group and on average 27.8 years (95% CI: 26.0–29.7) old with a BMI of 23.1 kg/m^2^ (95% CI: 21.6–24.7). Table 1 specifies the demographic data of the subjects. Vital signs and blood routine parameters were within normal range. Age was comparable in men and women and the significantly higher BMI of men in comparison to women (*p* = 0.0051) can be attributed to the difference in body composition. None of the subjects was vegetarian or vegan, and the majority of the participants (75%; *n* = 6 of each gender) stated doing sports regularly.

### 2.2. CBD Plasma Concentration Time Profile

**Figure 1:**
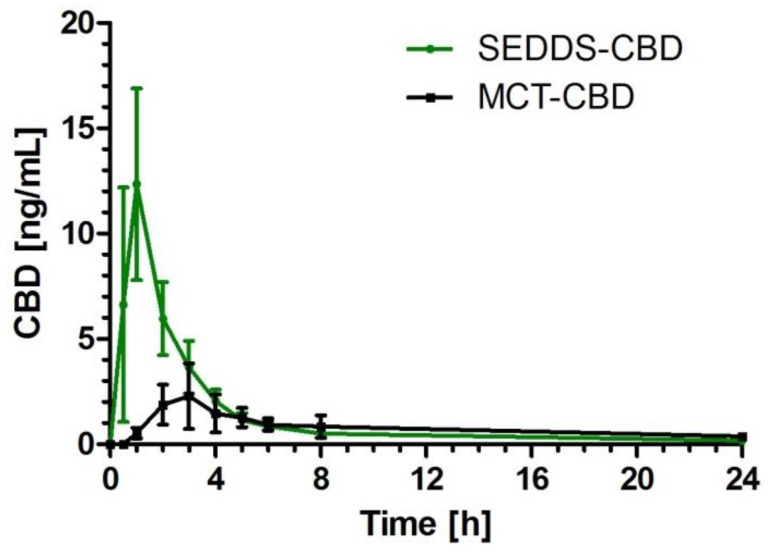
Cannabidiol (CBD) plasma concentration time profile after ingestion of self-emulsifying drug delivery system-cannabidiol (SEDDS-CBD) (green) and medium-chain triglycerides-cannabidiol (MCT-CBD) (black) depicted as summary curves of mean values at single time points (mean ± 95% CI) for all subjects.

After application of the study products, there was a significant increase of CBD plasma concentration over time in both formulations (*p* < 0.0001). For SEDDS-CBD the concentration was significantly increased starting from 0.5 h to 6 h compared to baseline levels. For MCT-CBD the concentration time curve was on a much lower level but also with significant increase of CBD concentration starting from 1 h to 8 h in comparison to baseline (Figure 1). After 8 h, most of the CBD was metabolized and/or eliminated, reaching nearly baseline levels. However, low concentrations could still be detected in all subjects after administration of both products.

### 2.3. Pharmacokinetic Parameters

The SEDDS-CBD formulation provided improved bioavailability over the MCT-CBD formulation as assessed by AUC. AUC_0–8h_ of SEDDS-CBD was 2.85-fold higher compared to MCT-CBD (*p* < 0.0001). The significant difference was also confirmed for AUC_0–24h_ (*p* = 0.0021) (Table 2).

C_max_ levels were significantly higher (>4-fold) after administration of SEDDS-CBD compared to the reference product (*p* < 0.0001) (Table 2). Furthermore, absorption of CBD from SEDDS-CBD was significantly faster compared to MCT-CBD (*p* < 0.0007). T_max_ was 1.0 h for SEDDS-CBD and 3.0 h for MCT-CBD (Table 2). Despite the observed high inter-individual variability of the bioavailability of CBD in both formulations with a coefficient of variation of 58.58% for SEDDS-CBD vs. 74.66% for the reference product (data presented for AUC_0–8h_), the very fast absorption of CBD from SEDDS-CBD with T_max_ values of ≤1 h was very homogeneously confirmed in 87.5% of subjects (maximum T_max_ levels 2 h).

The pharmacokinetic endpoints were additionally investigated for women and men separately. In both subgroups the significantly higher bioavailability and faster absorption of SEDDS-CBD in comparison to MCT-CBD were confirmed (Table 2).

Overall, assessment of 90% confidence interval (CI) for the ratio of geometric means further confirmed superiority of SEDDS-CBD compared to MCT-CBD for all evaluated pharmacokinetic parameters (90% CI > 1.25).

### 2.4. Effects of Gender on CBD Pharmacokinetics

**Figure 2:**
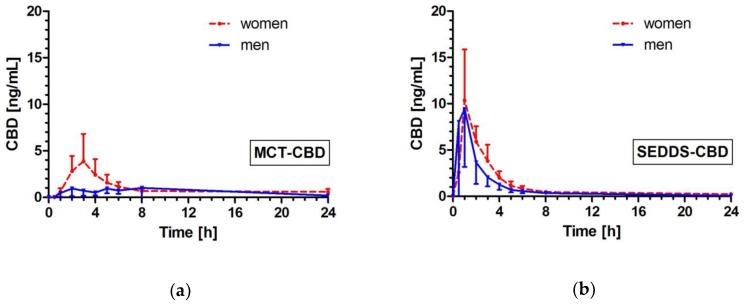
CBD plasma concentration time profile after ingestion of MCT-CBD (**a**) and SEDDS-CBD (**b**) depicted as summary curves of mean values at single time points (mean ± 95% CI) for women (red, dotted-line) and men (blue, solid line).

Comparison between men and women within the product groups indicated higher CBD bioavailability in women than in men (Figure 2). This difference was more pronounced for MCT-CBD with significant differences. For MCT-CBD, the AUC_0–8h_ and AUC_0–24h_ values were 2.4-fold (*p* = 0.0192) and 1.5-fold (*p* = 0.0499) higher in women than in men (Table 2). For MCT-CBD, women showed on average a 2.2-fold higher C_max_ value compared to men, but this did not reach statistical significance (*p* = 0.1080).

In contrast, after ingestion of SEDDS-CBD the effects of gender on bioavailability were minor with no significant differences and comparable C_max_ values (13.75 ng/mL (men) vs. 13.32 ng/mL (women) (*p* = 0.7209)). Only T_max_ was reached slightly faster in men in comparison to women after administration of SEDDS-CBD (*p* = 0.0341).

### 2.5. Safety Assessment

All subjects (100%) rated the tolerability of the study products as “well tolerated” during the kinetic days. Adverse events not related to study product noted within 24 h post-dosing included headache (in six subjects), nausea (in 1 subject) and menstrual cramps (in 1 subject). One adverse event was rated as unlikely related to study product by subject (diarrhea approximately 29 h after product ingestion). There were no serious adverse events.

## 3. Discussion

The interest and growing demand worldwide for natural cannabidiol (CBD) due to its many health benefits is clear. To insure the beneficial effects of this botanical extract we explored how to optimize its oral bioavailability and pharmacokinetics.

CBD shows limited oral bioavailability due to its poor aqueous solubility and extensive first-pass metabolism [13,14,19,30,31]. To overcome these limitations a novel self-emulsifying drug delivery system (SEDDS) based on VESIsorb^®^ formulation technology (SEDDS-CBD) was developed. The data presented show that SEDDS-CBD provided improved bioavailability over MCT-CBD. A single oral dose of SEDDS-CBD resulted in a 2.85-/1.70-fold increase in AUC_0–8h_/AUC_0–24h_ and a 4.4-fold increase in C_max_ compared to MCT-CBD.

Solubilization of CBD in the aqueous environment of the gastrointestinal (GI) tract is thought to be the main mechanism by which SEDDS-CBD is improving the oral bioavailability of CBD. The SEDDS-CBD mediated absorption pathway of CBD can be broken down as follows: (i) SEDDS-CBD spontaneously forms tiny droplets upon contact with the gastric or intestinal fluid. The formed droplets contain/solubilize the co-administered lipophilic CBD; (ii) after formation, the droplets diffuse across the aqueous lumen of GI-tract to the surface of the enterocyte; (iii) once at the surface of the enterocyte, single molecules of CBD dissociate from the droplets and are transferred to the enterocyte membrane (flip-flop) and further to the blood vessels.

In a recently performed systematic search of PubMed and EMBASE (including MEDLINE), with the aim to review and analyze all available pharmacokinetic data of CBD, it is illustrated that the pharmacokinetic profile of CBD is dependent on the route of application, dosage schedule (single, multiple dose), type of formulation system and diet (fasted vs. fed). Of 792 articles retrieved, 24 included pharmacokinetic parameters in humans and were summarized and compared [15]. The review article cites two studies using SEDDS as delivery system: the pro-nano-dispersion technology (PTL401, Atsmon et al. [22]) and the piperine-pro-nanolipospheres (PNL, Cherniakov et al. [23]), the latter combining the SEDDS formulation principle (solubilisation) with the absorption enhancer piperine. Piperine, naturally found in black pepper, has been shown to inhibit first pass metabolism mechanisms such as Cytochrome P450 family enzymes and the P-glycoprotein efflux pump [23]. Thus, the PNL formulation may increase the bioavailability of cannabinoids CBD and THC not only by increasing their solubility in the gastrointestinal tract, but also by inhibiting or reducing their first pass metabolism.

The AUC data of SEDDS-CBD compare favorable to both SEDDS formulations, PNL and PTL401. The AUC_0–24h_ value of SEDDS-CBD was 1.9- and 1.3-fold higher compared to PNL and PTL401, respectively (PNL: AUC_0–24h_ 6.9 ng/mL*h, 10 mg dose, fasted; PTL401: AUC_0–24h_ 9.85 ng/mL*h, 10 mg dose, fed; SEDDS-CBD: AUC_0–24h_ 13.1 ng/mL*h, 10 mg dose-adjusted, fasted).

Furthermore, the C_max_ values of SEDDS-CBD were 2.6- and 1.9-fold higher compared to PNL and PTL401, respectively (PNL: C_max_ 2.1 ng/mL, 10 mg dose, fasted; PTL401: C_max_ 2.9 ng/mL, 10 mg dose, fed; SEDDS-CBD: C_max_ 5.4 ng/mL, 10 mg dose-adjusted, fasted).

We are aware that inter-study comparisons have to be made with caution and therefore the following comments should be considered:

First, the CBD dose of the current study was 25 mg whereas the CBD dose administered by Cherniakov et al. (PNL) [23] and Atsmon et al. (PTL401) [22] was 10 mg. An approximately dose-proportional increase in AUC after the administration of single dose of 10 mg and 20 mg CBD has been shown by Stott et al. [32]. A saturation effect was observed at CBD doses of 400–800 mg [15]. Thus, the dose-adjusted comparison of the current test product with PNL and PTL401 is certainly reasonable.

Second, the current study and the study by Cherniakov et al. [23] were conducted under fasted conditions whereas the study by Atsmon et al. [22] was carried out under fed conditions. The relevance of these dietary conditions for CBD’s pharmacokinetics was recently demonstrated by Stott et al. who reported an increase in CBD bioavailability under fed vs. fasted states in 12 men after a single 10 mg dose of CBD administered as oromucosal spray (Sativex^®^) [16]. Mean AUC and C_max_ were 4- and 3-fold higher during fed compared to fasted conditions (AUC_0–t_, 20.2 vs. 4.5 ng/mL*h; C_max_ 3.7 vs. 1.2 ng/mL). Absorption was delayed in the fed state (T_max_ 4.0 vs. 1.4 h). A 4-fold increase of AUC levels has also been reported for Epidiolex^®^, when administered with a high-fat/high-caloric meal [33]. Epidiolex^®^, recently approved by the FDA for the treatment of rare childhood-onset epileptic seizures, is an oral oily solution. The formulation excipients include sesame oil, ethanol and flavours [33]. The possible impact of food on bioavailability of the current study product cannot be estimated from the study data and should be assessed in further studies to allow comparison to studies reporting CBD bioavailability under fed conditions.

Third, the oromucosal spray Sativex^®^ was used as a reference product by Cherniakov et al. [23] and by Atsmon et al. [22]. As Sativex^®^ is classified as a pharmaceutical product additionally containing THC, the product could not be used as reference in the current study. Anyhow, the pharmacokinetic data of SEDDS-CBD compare favorable to Sativex^®^, a solution of CBD/THC in ethanol/propylene glycol. The AUC_0–24h_ value of SEDDS-CBD was 4.2-fold higher compared to Cherniakov et al. (fasted) and 1.8-fold higher compared to Atsmon et al. (fed) (Sativex^®^: AUC_0–24h_ 3.1 ng/mL*h, 10 mg dose, fasted; Sativex^®^: AUC_0–24h_ 7.3 ng/mL*h, 10 mg dose, fed; SEDDS-CBD: AUC_0–24h_ 13.1 ng/mL*h, 10 mg dose-adjusted, fasted).

In the current study, absorption of CBD from SEDDS-CBD was substantially faster compared to the reference with 87.5% of subjects showing peak concentration within 1 h, whereas the median of T_max_ for the MCT-CBD was 3.0 h. A fast absorption of CBD is favorable in various conditions, especially in the therapeutic field. With respect to data presented in literature, T_max_ values for SEDDS-CBD and PNL were comparable (PNL: T_max_ 1.0 h vs. SEDDS-CBD: T_max_ 1.0 h) under fasted conditions. In contrast, PTL401 administered under fed conditions showed a slightly higher T_max_ value of 1.64 h [22]. Such a shift of T_max_ values was also reported by Stott et al. comparing the concentration time profile of Sativex^®^ under fasting and fed conditions [16]. T_max_ values were 1.4 h and 4.00 h for the fasted and fed state, respectively. The delayed absorption of CBD under fed conditions might be explained at least partially by the increased gastric transit time after consumption of meals with high fat content.

The pharmacokinetic endpoints were additionally investigated separately for women and men. Differences in AUC and C_max_ were observed between women and men after ingestion of MCT-CBD with a higher absorption in women compared with men. A weak correlation was seen in the current study between AUC_0-8h_ and body weight (r = −0.4260, *p* = 0.0999) after ingestion of MCT-CBD, but not with BMI. However, gender differences have been reported for all phases of compound disposition (absorption, distribution, metabolism, excretion) and may be due to molecular and physiological factors. The molecular factors include the metabolism rate of cytochrome P-450 (CYP) enzymes and relevant transporter systems [34,35,36]. Physiological factors comprise not only the lower body weight of females, but might be also ascribed to other intrinsic factors such as differences in distribution volume, higher percentage of body fat, lower glomerular filtration rate, slower gastric motility, or hormonal status of women [37,38,39,40]. Nadulski et al. also reported differences between men and women with significantly higher AUC and C_max_ values found for females as compared with males after oral application of THC or CBD [41] and thus confirmed the current findings. In this context, it is worth mentioning that the “one size fits all” dosage strategy often leads to higher exposures in women also for other substances [42].

Interestingly, the gender differences were much less pronounced for SEDDS-CBD. The correlation with body weight was overrode (r = −0.1457, *p* = 0.5903). SEDDS-CBD seems to overcome the physiologic responsible differences in women and men due to the enhanced delivery of cannabidiol. However, these observations are based so far on a very limited sample size of *n* = 8 and should be confirmed in further studies. Since these gender differences are important and the reasons/mechanisms behind this warrant further research to truly understand further.

With regards to study limitations, we like to address the high inter-individual variability of CBD bioavailability which accounted for 60.5% after MCT-CBD and 54.4% after SEDDS-CBD administration (based on AUC_0–24h_). Such high inter-individual variability was already reported in previous literature [10,17,18,31]. Therefore, the study was performed in cross-over design to control for inter-individual variability. As study products were only provided once to subjects, intra-individual variability cannot be estimated from the data. Anyhow, the inter-individual differences should be taken into consideration in the clinical setting. The possible impact of food on bioavailability of SEDDS-CBD cannot be estimated from the study data and should possibly be assessed in further studies.

As data continue to emerge looking at the efficacy of CBD on various functional outcomes, achieving certain thresholds for plasma levels of CBD may be important. Although this study did not investigate efficacy, it is possible that a product’s efficacy or lack thereof, may be correlated with its ability to deliver sufficient CBD to the blood.

## 4. Materials and Methods

### 4.1. Study Subjects

**Figure 3:**
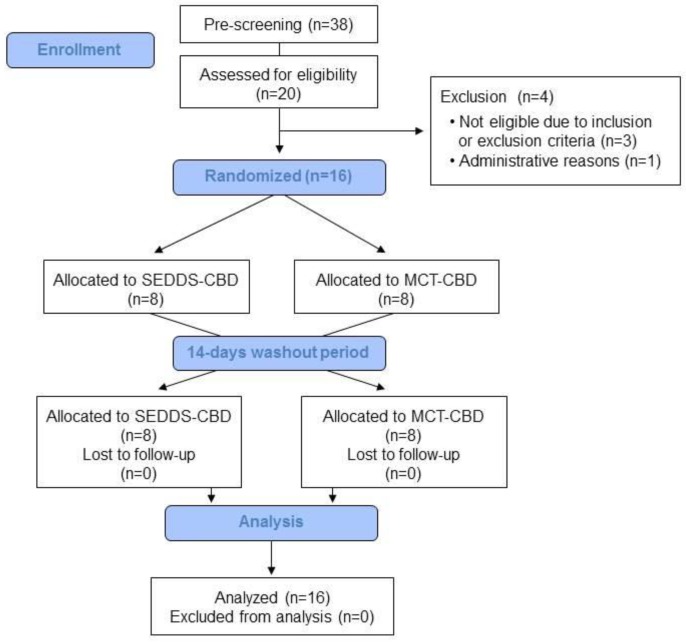
Study flow.

Between beginning of October 2018 and mid of November 2018 a total of 38 subjects were pre-screened for their eligibility and thereof 20 subjects invited for screening visit as shown in Figure 3. According to inclusion criteria, subjects had to be aged between 18–50 years with a body mass index (BMI) of 19–30 kg/m^2^ and non-smoking. Furthermore, subjects had to be in good physical and mental health as established by the medical history, physical examination, electrocardiogram, vital signs, results of biochemistry and hematology.

The main exclusion criteria were a relevant history or presence of any medical disorder, potentially interfering with this study (e.g., mal absorption, chronic gastrointestinal diseases, heavy depression, cardiovascular disease event within last 3 months, etc.), use of hemp/cannabis products at least 1 week prior to study start to exclude possible interaction, regular intake of drugs or supplements possibly interfering with this study, and drug-, alcohol- and medication abuses. Medications for treatment of chronic diseases that do not affect the metabolism of the study product were permitted and were judged individually regarding interference with study by investigator. Any concomitant chronic medication and medication used for the treatment of adverse events (AEs) was documented. Reasons for non-inclusion were low hemoglobin levels (3x) and schedule difficulties. Finally, 16 subjects (8 men, 8 women) were included and all completed the study successfully without considerable protocol deviations.

This study was conducted in orientation towards the guidelines of the Declaration of Helsinki and Good Clinical Practice. The protocol and all documents were approved by the Institutional Review Board (IRB) of Landesärztekammer Baden-Württemberg with the reference number F-2018-049. Written informed Consent Form was obtained from all participants prior to screening evaluations. The study was registered in the German Clinical Trials Register (DRKS00015283).

### 4.2. Study Design

The clinical study was performed as a randomized, double-blind, monocentric and controlled cross-over design at the study site of BioTeSys GmbH (Esslingen, Germany). 16 healthy volunteers (50% of each gender) were randomized to receive on separate kinetic days a single dose of Hemp Extract (standardized to 25 mg CBD) as either SEDDS-CBD or MCT-CBD together with 250 mL of still water under fasted conditions. On each dosing occasion, subjects fasted for at least 10 h overnight prior to and 4 h post dosing. There was a washout period of 14 days between each kinetic day. Each treatment period consisted of an in-clinic stay until blood sampling 8 h post-dosing and a subsequent visit for the 24 h follow-up. Blood samples were collected at pre-dosing and 0.5, 1, 2, 3, 4, 5, 6, 8 and 24 h after product administration for CBD analysis using liquid chromatography-mass spectrometry (LC-MS/MS) technique. Dinner prior to kinetic days as well as all meals and fluid intake were standardized until 24 h post-dosing. Meals were served during kinetic days at 4, 7, 10, and 13 h post administration of study products. Furthermore, subjects were asked to avoid alcohol 24 h and flaxseeds and flaxseed oil 48 h before study visits. No strenuous physical activity or endurance sports were allowed within 24 h prior to study visits.

During the study intervention, the subjects documented any adverse events and concomitant medication in diaries. The overall tolerability was assessed at the end of each kinetic day.

### 4.3. Intervention

The plant part used were aerial parts of Cannabis sativa L. (hemp), and the respective extract was a concentrated phytocannabinoid extract with a CBD content of 60% and free of THC (≤0.05%). For the investigational product the Hemp-Extract was formulated as self-emulsifying drug delivery system (SEDDS) based on proprietary VESIsorb^®^ formulation technology, comprising food emulsifiers, edible vegetable oils and fatty acids, further referred as “SEDDS-CBD”. The SEDDS-CBD formulation was characterized by measuring the size of the droplets formed upon dilution with purified water at 37 °C (1 weight part of SEDDS-CBD was diluted with 99 parts of water). The droplet size was determined using a Zetasizer Nano S instrument (Malvern Instruments Limited, Worcestershire, UK). The mean diameter of the droplets formed is between 40 to 50 nm. The size distribution of the droplets is homogeneous exhibiting one main population (Polydispersity index <0.100). The storage stability of SEDDS-CBD is given for at least six months at 25 °C as assessed by droplet formation. As reference formulation the same Hemp-Extract diluted with MCT oil, further referred as “MCT-CBD” was used. Both formulations were filled into colored, vegetarian, liquid-filled hard-shell capsules delivering 25 mg CBD per capsule. Manufacturing and encapsulation were carried out in compliance with GMP conditions and all excipients as well as capsule shell met the current European food regulations. To ensure double-blind conditions investigational (SEDDS-CBD) and reference (MCT-CBD) product looked fully identical regarding size, color, odor and secondary packaging. Capsules were provided by Vesifact AG (Baar, Switzerland).

### 4.4. Sample Analysis

Blood samples for safety parameters (differentiated hematogram and clinical laboratory including lipid status and liver enzymes) were collected at screening and during kinetic days at pre-dosing as well as 24 h post-dosing. Safety parameters were performed at an accredited lab (Synlab Medizinisches Versorgungszentrum Leinfelden-Echterdingen, Germany) the same day. For determination of CBD plasma concentration venous blood was collected in EDTA monovettes (Sarstedt, Germany). Blood samples were processed under light-protected condition and were centrifuged at 3000× *g* for 10 min at 4 °C. Processing time was below 30 min until freezing at −80 °C of plasma aliquots.

Determination of CBD plasma concentration was performed by LC-MS/MS in electrospray ionization (ESI) multiple reaction monitoring (MRM) mode (LC/MS/MS XEVO TQ-S Micro). Detailed LC-MS/MS conditions are listed in Table S1. 50 µL internal standard (d3-CBD, 20 ng/mL in methanol) was added to 200 µL plasma samples. CBD was extracted by liquid/liquid extraction using a mixture of diethylether/ethanol (6:1). After mixing and centrifugation the upper phase was transferred in a glass vial and evaporated under nitrogen. The residue was reconstituted with 100 µL 50% acetonitrile/water. 10 µL of supernatant was injected to a Waters Acquity BEH C18, 2.1 × 50 mm, 1.7 µm UPLC column at 40 °C with 0.50 mL/min flow rate. The eluents A (water), B (0.1% (*v*/*v*) formic acid in methanol) and C (acetonitrile) were used in a gradient elution of holding initially 30% A/70% B for one minute, followed by a linear gradient to 80% B/20% C within 2.5 min. The composition was retained for another minute and the column reequilibrated with the initially eluent composition. Interassay precision was 7.6%, and limit of quantification was 0.25 ng/mL.

### 4.5. Analysis Software and Statistical Analysis

Pharmacokinetic parameters were calculated individually using the blood concentration-time curves. Area under the observed concentration-time curve above baseline (AUC), more precisely AUC_0–8h_ and AUC_0–24h_, was calculated applying the trapezoidal rule with the y-axis, defined by CBD plasma concentration, and the x-axis defined via sampling time points. Plasma concentrations of the blood samples below the lower limit of quantification at early and late time points were treated as zero. Curve progression was analyzed by Friedman test. Peak plasma concentration after administration (C_max_) and time to reach C_max_ (T_max_) were adequately calculated. For data analysis AUC_0–8h_, AUC_0–24h_ and C_max_ were log-transformed. After log transformation AUC_0–8h_, AUC_0–24h_ and C_max_ were calculated using a linear mixed model taking into account sequence, period and product. Differences between T_max_ were evaluated by Wilcoxon rank sum test. All 16 subjects were included in the analysis. Statistical tests were performed two-sided and *p* values <0.05 were statistically significant. AUC and C_max_ are presented as mean ±95% confidence interval (CI) and T_max_ as median with 25th–75th percentile. Statistical evaluation, summary tables and graphs were generated using GraphPad Prism software (La Jolla, CA, USA) and SAS V9.3 statistical software (SAS Institute, Cary, North Carolina).

## 5. Conclusions

The objective of the current study was to assess whether a novel SEDDS formulated Hemp-Extract based on VESIsorb^®^ formulation technology (SEDDS-CBD) may improve the oral bioavailability of CBD compared to MCT formulation (MCT-CBD) in healthy subjects. The bioavailability measured as AUC and C_max_ was significantly higher and CBD was absorbed significantly faster in comparison to the reference product. To conclude, SEDDS-CBD based on VESIsorb^®^ formulation technology offers a novel, good, tolerable, and effective oral cannabinoid delivery system. CBD has a number of potential health benefits, however, our data demonstrated that unless the SEDDS formulation is used, there is relatively poor bioavailability of the standard CBD formulations (e.g., Hemp-Extract diluted with MCT oil) and could lead to diminished benefits (or no benefit) for this natural product. Thus, it has to be considered that a significant health benefit always stays in relation to the bioavailability of a product.

## Figures and Tables

**Table 1:** Demographic and Baseline Data.

Variable	Men		Women	
	Mean	95% CI	Mean	95% CI
**Age (years)**	27.8	(23.7–31.8)	27.9	(26.4–29.3)
**BMI (kg/m^2^)**	25.0	(23.4–26.5)	21.3	(19.1–23.4)
**Systolic BP (mmHg)**	129.1	(121.5–136.7)	118.6	(109.1–128.1)
**Diastolic BP (mmHg)**	76.9	(73.2–80.5)	73.1	(65.5–80.7)
**Hemoglobin (g/dL)**	15.3	(14.1–16.1)	13.0	(12.3–13.6)
**Total Cholesterol (mg/dL)**	171.8	(148.0–195.5)	180.3	(156.5–204.0)

BMI: body mass index; BP: blood pressure; CI: confidence interval.

**Table 2:** Pharmacokinetic Parameters for self-emulsifying drug delivery system-cannabidiol (SEDDS-CBD) and medium-chain triglycerides-cannabidiol (MCT-CBD).

Subjects	Pharmacokinetic Parameters											
	C_max_ [ng/mL]			AUC_0-8h_ [ng/mL*h]			AUC_0-24h_ [ng/mL*h]			T_max_ [h]		
	Mean (95% CI)			Mean (95% CI)			Mean (95% CI)			Median (25^th^–75^th^ percentile)		
	MCT-CBD	SEDDS-CBD	*p*	MCT-CBD	SEDDS-CBD	*p*	MCT-CBD	SEDDS-CBD	*p*	MCT-CBD	SEDDS-CBD	*p*
All (*n* = 16)	3.05 (1.57–4.54)	13.53 (7.96–19.10)	<0.0001	9.51 (5.73–13.30)	27.15 (18.68–35.63)	<0.0001	19.23 (13.03–25.42)	32.63 (23.18–42.08)	0.0021	3.0 (2.0–5.0)	1.0 (1.0–1.0)	0.0007
Men (*n* = 8)	1.93 (0.89–2.96)	13.75 (2.83–24.68)	0.0013	5.54 (3.87–7.22)	24.86 (8.05–41.66)	0.0005	15.10 (4.51–25.70)	28.95 (10.68–47.22)	0.0112	4.0 (2.0–5.0)	1.0 (0.5–1.0)	0.0202
Women (*n* = 8)	4.18 (1.25–7.11)	13.32 (6.66–19.97)	0.0033	13.48 (6.59–20.38)	29.44 (20.06–38.82)	0.0095	23.35 (15.40–31.29)	36.31 (25.56–47.06)	0.0042	2.5 (2.0–3.0)	1.0 (1.0–1.8)	0.0187

## Supplementary Materials

The following are available online at https://www.mdpi.com/1420-3049/24/16/2967/s1.
