# Supplementary material for: A Novel Self-Emulsifying Drug Delivery System (SEDDS) Based on VESIsorb® Formulation Technology Improving the Oral Bioavailability of Cannabidiol in Healthy Subjects
Source: Molecules. 2019 Aug 16;24(16):2967. doi: 10.3390/molecules24162967 (PMC6720748; doi:10.3390/molecules24162967)
Supplement: Supplementary file 1 [file molecules-24-02967-s001.pdf]

Table S1: MRM settings of the final ESI +-MS/MS method.

Analyte	Parent ion (m/z)	Daughter ion (m/z)	Dwell time [s]	Cone voltage [V]	Collision energy [V]	Ion transition type
CBD	315.38	93.01	0.011	18	24	q
	315.38	123.00	0.011	18	32	q
	315.38	193.10	0.011	18	20	Q
IS	318.41	196.17	0.025	34	22	Q
	318.41	123.06	0.025	44	32	q

LC-MS/MS parameters: WATERS XEVO-TQS micro in ESI + mode; Capillary voltage: 1.9 kV; Source temperature: 400°C; Desolvation gas flow: 1000 L/h; Cone gas flow: 50 L/h: Recorded channels: MS2 scan 50-1000 m/z mass units, 0.1 s scan time. Q: Quantifier ion transition, q: qualifier ion transition
